# Hematopoietic stem cell transplantation for CYBB heterozygous mutation resulting in very early onset inflammatory bowel disease in children: a case report

**DOI:** 10.1186/s12887-023-04158-z

**Published:** 2023-07-11

**Authors:** Zhiling Li, Huan Chen, Xiaoqin Feng, Yongsheng Ruan, Min Yang

**Affiliations:** 1 Department of Pediatrics, Guangdong Provincial People’s Hospital (Guangdong Academy of Medical Sciences), Southern Medical University, Guangzhou, Guangdong China; 2 Department of Pediatrics, Guangzhou Women And Children’s Medical Center, National Children’s Medical Center For South Central Region, Guangzhou, Guangdong China; 3 Department of Pediatrics, Nanfang Hospital, Southern Medical University, Guangzhou, Guangdong China

**Keywords:** Hematopoietic stem cell transplantation, Gene mutations, *CYBB*, Very early-onset inflammatory bowel disease, Dihydrorhodamine assay, Children

## Abstract

**Background:**

Inflammatory bowel disease (IBD) is a heterogeneous group of disorders associated with environmental triggers and dysregulated immune responses resulting in chronic, recurrent intestinal inflammation. Very early-onset IBD (VEO-IBD) refers to patients with symptoms or diagnosis before the age of 6 years and is widely thought to be associated with monogenic mutations. Traditional drug therapy is often ineffective in this patient population, while hematopoietic stem cell transplantation (HSCT) represents the definitive cure for patients with gene mutations.

**Case presentation:**

We report a case of VEO-IBD associated with a monogenic mutation in a 2-year-old girl presenting mainly with gastrointestinal symptoms, including recurrent hematochezia and abdominal pain for more than 3 months. A gastroscopy revealed erosive gastritis and bulbar duodenitis, while a colonoscopy indicated erosive colitis. Abnormal results were obtained from the dihydrohodamine (DHR) assay and immunoglobulin testing. Whole-exome sequencing identified a heterozygous and de novo nonsense mutation (c.388 C > T; p.R130X) in the *CYBB* gene leading to deficiency of nicotinamide adenine dinucleotide phosphate (NADPH) oxidase 2 (NOX2) (encoded by *CYBB*), a critical component of phagocytes. HSCT was performed successfully, and the DHR assay showed that normal neutrophil function was restored. Six months after HSCT, clinical remission was observed, and a repeat colonoscopy revealed intestinal mucosal healing was attained.

**Conclusions:**

Patients with *CYBB* mutations often develop recurrent or severe bacterial or fungal infections, mostly in the lungs, skin, lymph nodes, and liver. Here, we report on a young female child with *CYBB* mutations presenting predominantly with gastrointestinal symptoms. This study explores the mechanisms of inflammatory bowel disease caused by a monogenic mutation in *CYBB* to improve early diagnosis and effective treatment rates of this patient population.

## Background

Inflammatory bowel disease (IBD) is a group of chronic, non-specific intestinal inflammatory diseases that can occur at any age with unknown etiology. IBD can be divided into ulcerative colitis (UC) and Crohn’s disease (CD). UC mainly involves the colorectal mucosa, presenting with diarrhea, mucous and bloody stools, and abdominal pain. CD can involve any part of the digestive tract from the mouth to the anus, manifesting as abdominal pain, diarrhea, and anal lesions. Very early-onset IBD (VEO-IBD) in patients with symptoms before 2 years are more likely to be associated with monogenic mutations that alter immune function and present with more severe disease [1]. Due to the abundance of variants in primary immunodeficiency genes in patients with VEO-IBD, some genes involved in immunodeficiency cause severe intestinal disease and systemic autoimmunity [2]. Several monogenic mutations have been identified in children with VEO-IBD, including IL‐10, IL‐10RA/B, XIAP, and TTC37 [3]. The patients often respond poorly to standard therapies, including biological agents [4]. Hematopoietic stem cell transplantation (HSCT) represents a definitive cure for diseases associated with gene mutations. For individuals indicated for HSCT, active infections should be treated prior to transplantation due to the increased risk of mortality.

*CYBB* (Cytochrome B-245 Beta Chain) is a protein-coding gene, and its mutation results in the deficiency of NADPH oxidase 2 (encoded by *CYBB*), which form the complex of nicotinamide adenine dinucleotide phosphate (NADPH) in phagocytes. NADPH represents a source of reducing equivalents for neutrophil respiratory burst oxidase [5]. Current evidence suggests that NADPH defects cause respiratory burst dysfunction in phagocytes (neutrophils, monocytes and macrophages), leading to the inability to produce reactive oxygen species (ROS), which activate granule proteases to destruct phagocytosed microorganisms. The dihydrorhodamine (DHR) flow cytometry assay is a useful diagnostic tool that can detect absent or reduced NADPH oxidase activity in stimulated phagocytes. Defective production of ROS leads to increased expression of nuclear factor (NF) kappa-B-regulated inflammatory genes, the hyperactivation of NF-ĸB and inflammasome in phagocytes lead to long-lasting production of pro-inflammatory cytokines and inflammatory manifestations, such as inflammatory bowel disease [6].

In this report, we identified a heterozygous and de novo nonsense mutation in the *CYBB* gene in a 2-year-old girl with IBD features. *CYBB* mutations are usually associated with X-linked chronic granulomatosis (X-CGD). Unlike most patients with this disease presenting with recurrent bacterial or fungal infections, our patient mainly exhibited gastrointestinal symptoms, such as hematochezia and abdominal pain. This report provides preliminary evidence of a de novo mutation (c.388 C > T; p.R130X) in the *CYBB* gene as a new disease-causing mechanism for VEO-IBD.

## Case presentation

A 28-month-old girl was admitted to a local medical institution due to repeated hematochezia for more than 3 months. She experienced hematochezia for no obvious reasons at the age of 25 months, with small to moderate amounts of dark red or bright red paste-like stool 3–5 times a day, accompanied by abdominal pain, mainly around the umbilical area, which had no obvious relationship with eating and bowel function, and was not accompanied by vomiting, diarrhea, fever, and cough. Routine stool analysis showed red and white blood cells, but the pathological culture was negative. The complete blood count indicated that the white blood cell count was 16.3 × 10^9^/L, and the hemoglobin concentration was 123 g/L. Abdominal ultrasound showed active intestinal peristalsis, multiple hypoechoic nodules around the umbilical area, and enlarged mesenteric lymph nodes. Bacterial enteritis was suspected and treated with anti-infection and hemostatic drugs. During the period, she was fed with deeply hydrolyzed formula which yielded poor therapeutic effects. Eventually, the patient was transferred to our hospital.

The patient was born to a healthy and non-consanguineous Chinese couple after a normal pregnancy with an unremarkable family history. The patient was delivered naturally and was generally in good condition after delivery. The patient previously underwent a cervical lymph node abscess biopsy at a local hospital, but the parents could provide no relevant clinical documentation. No abnormalities in growth and development were observed.

**Physical and Laboratory examination** During the physical examination upon admission, a small rash was observed on the neck. The complete blood count revealed a white blood cell count of 11.70 × 10^9^/L (reference range: 5.0–9.0 × 10^9^/L), hemoglobin 110 g/L (105–145 g/L), platelet count 38 × 10^9^/L (140–440 × 10^9^/L), and neutrophil percentage 31% (40-60%). Serum immunoglobulin testing revealed an immunoglobulin G (IgG) of 16.7 g/L (3.83 ~ 10.58 g/L), IgM 1.66 g/L (0.4 ~ 1.28 g/L), and IgE 74 IU/ML (0 ~ 60IU/ML). The absolute counts of B cells, T cells and NK cells were 1688.52 cells/ul (90–660 cells/ul), 4833.95 cells/ul (690–2540 cells/ul), and 877.12 cells/ul (90–590 cells/ul), respectively. Antiprotease 3 antibody (ELISA) 64.5 (less than 18). Food allergen IgE: milk 1.59 (less than 0.35 IU/ml). C-reactive protein, procalcitonin, blood gas analysis, biochemical indexes, coagulation index, autoimmune antibody, rheumatoid factor, and T-SPOT.TB test yielded no abnormal findings. No abnormality was found on chest and abdominal radiographs.

**Fig. 1:**
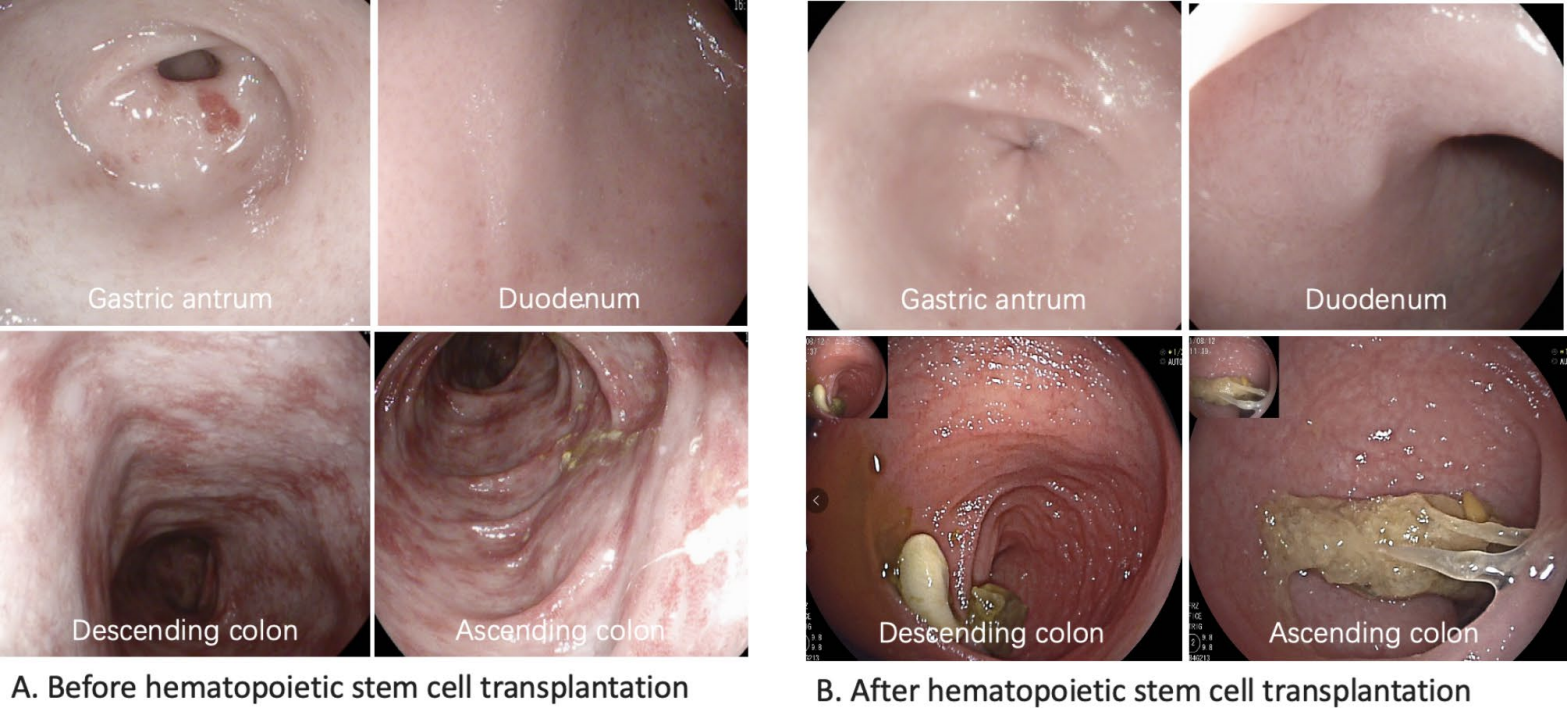
The represented endoscopy images before and after hematopoietic stem cell transplantation. (A) Endoscopy images characteristics as mucosal erythema and erosions in gastric antrum and colon before hematopoietic stem cell transplantation. (B) Endoscopy images showed gastric antrum and colonic mucosa erythema and erosion disappear after hematopoietic stem cell transplantation

**Fig. 2:**
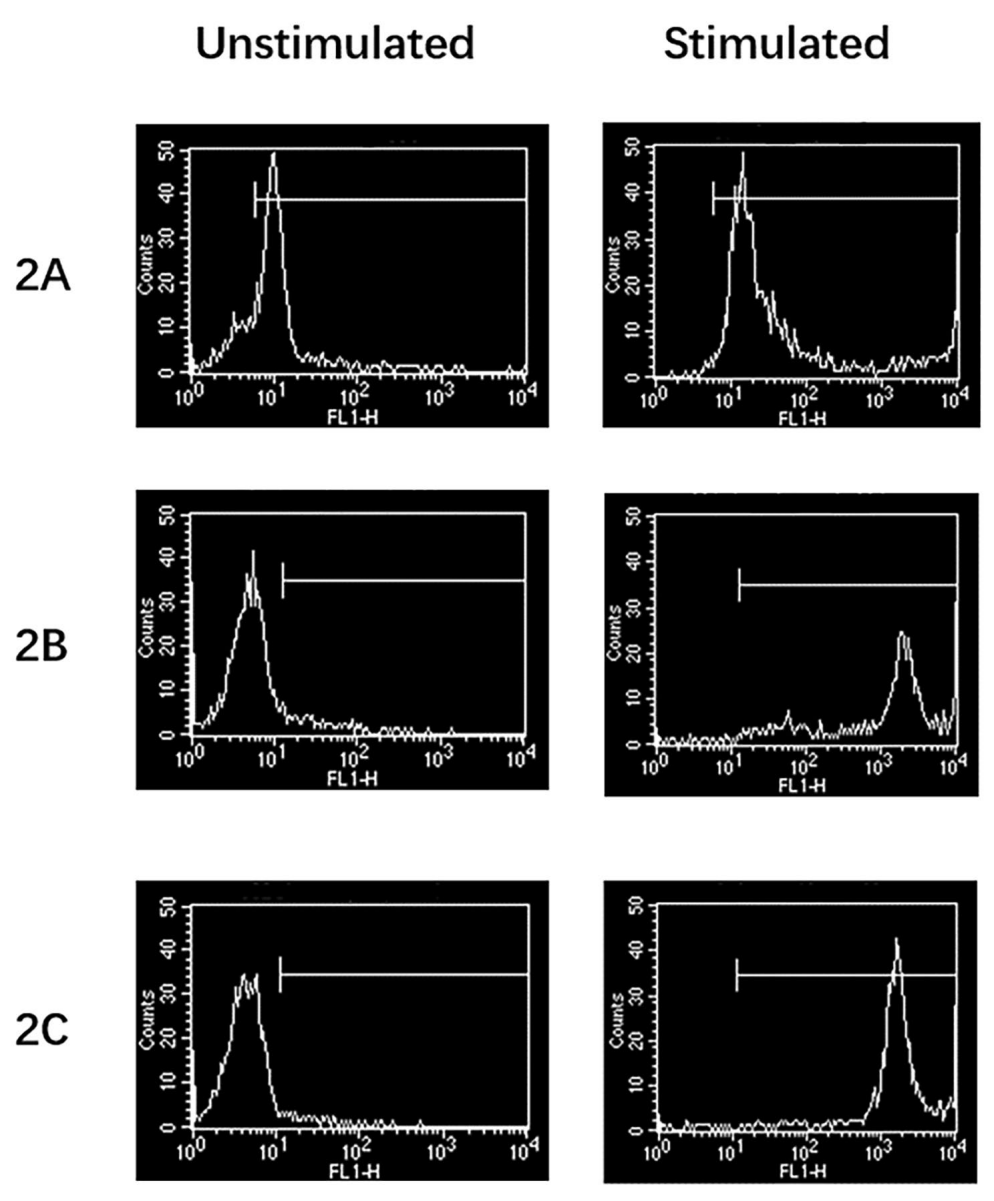
DHR histogram of the patient and his parents’ granulocytes. The histogram and stimulation index (SI) of the patient’s granulocytes (2 A) demonstrated a typical X-CGD pattern. The DHR of her mother’s granulocytes (2B) and her father’s granulocytes (2 C) demonstrated a normal histogram

**Fig. 3:**
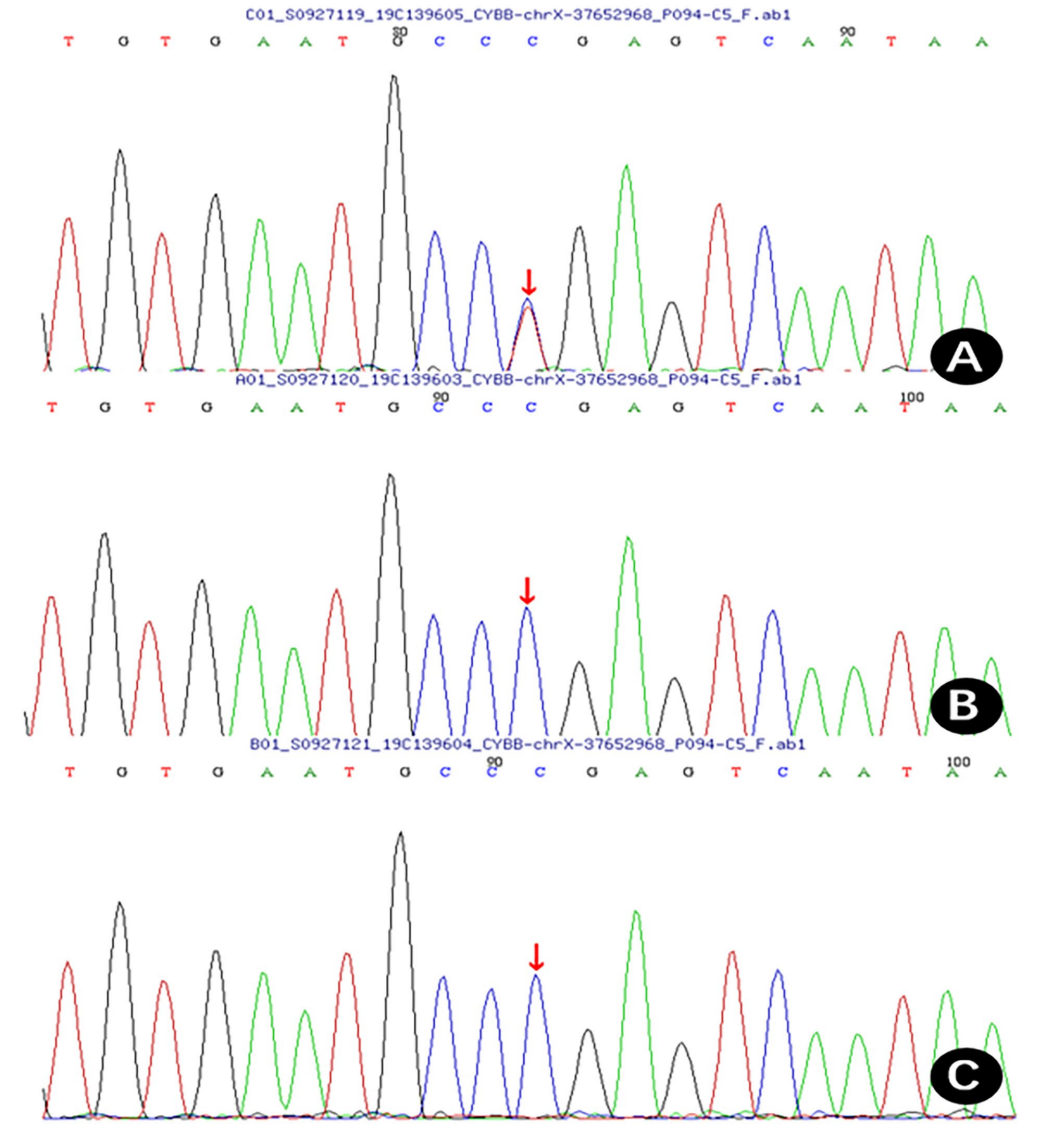
*CYBB* gene sequencing and parental verification results of child A: There was a heterozygous mutation of c.388 C > T in the *CYBB* gene (p.R130X), which was a de novo mutation. B (father of the child) and C (mother of the child) revealed no variation during sequencing. Arrows indicate mutation sites

**Fig. 4:**
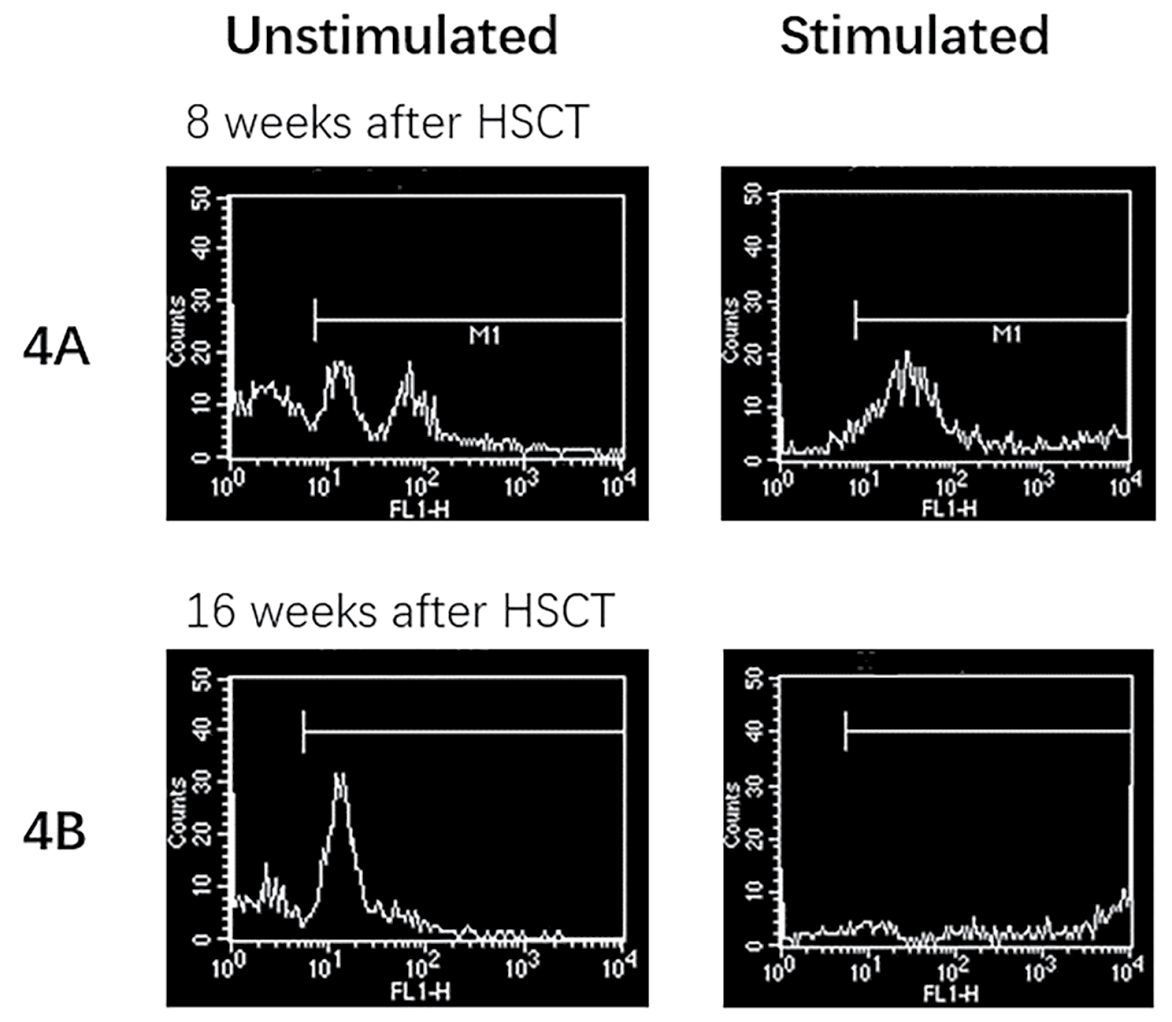
DHR assay after HSCT. DHR assay of granulocytes from the patient at 8 weeks after HSCT (4 A) showed abnormal histogram with the SI of 28.85, 16 weeks after HSCT (4B) showed normal histogram with the SI of 408.90, the normal neutrophil function was restored

**Diagnosis and treatment process and follow-up** Based on the medical history and test results, an initial diagnosis of cow milk protein allergy and congenital immunodeficiency disease was established. A gastroscopy was conducted and indicated erosive gastritis and erosive bulbar duodenitis. The gastric antrum and duodenal bulbar mucosa were biopsied, and the histopathological results showed mild chronic inflammation. A colonoscopy indicated erosive colitis (Fig. 1A). Pathological examination of mucosal surface tissues of each segment of the colon showed that the mucosa presented mild chronic inflammation, a large number of lymphocytes and eosinophils infiltrated in lamina propria, with a maximum of 85 cells per high power field, and crypt abscesses in the ascending and transverse colon. Next, the neutrophil oxidative burst test was performed using the DHR assay. The DHR assay of the patient’s granulocytes (Fig. 2A) revealed almost absence of fluorescence upon granulocyte stimulation. The stimulation index (SI) was 5.82 which was compatible with X-CGD. The DHR assay of granulocytes from the patient’s mother (Fig. 2B) and the patient’s father (Fig. 2C) showed normal histogram with the SI of 277.05 and 364.03, respectively. Therefore, peripheral venous blood was collected from the patient and her parents for whole exome sequencing analysis. A heterozygous de novo mutation c.388 C > T (p.R130X) in *CYBB* gene (Fig. 3) was identified and validated by Sanger sequencing. Based on the above findings, we established a diagnosis of very early onset inflammatory bowel disease (VEO-IBD) with neutrophil dysfunction caused by *CYBB* gene mutation. Hematopoietic stem cell transplantation (HSCT) was conducted using peripheral blood stem cells (20.47 × 10^8^/kg) from the father of the child. Mycophenolate Mofetil combined with Tacrolimus Capsules were used to prevent graft-versus-host disease (GVHD) and Voriconazole against fungal infections. Two months after HSCT, bone marrow and peripheral blood chimerism rates were 100% complete donor type, DHR assay of granulocytes from the patient at 8 weeks after HSCT (Fig. 4A) showed abnormal histogram with the SI of 28.85, 16 weeks after HSCT (Fig. 4B) showed normal histogram with the SI of 408.90, the normal neutrophil function was restored. A repeat colonoscopy six months after HSCT (Fig. 1B) showed complete intestinal mucosal healing. After 18 months of follow-up, there was no severe infection or acute or chronic GVHD-related manifestations.

## Discussion and conclusions

In this case, the heterozygous and de novo mutation c.388 C > T(p.R130X)was located in the second exon of *CYBB* gene and generated a premature stop codon, which was determined to be a pathogenic variant according to American College of Medical Genetics and Genomics guidelines. *CYBB* encodes the gp91 subunit of NADPH oxidase, and mutations impair the respiratory burst of all types of phagocytes [7]. The mutation was absent from her parents. and her parents had the normal neutrophil function. No mutation could be found on the patient’s other *CYBB* allele. Nevertheless, we had to consider two possibilities: The patient could have been compound heterozygous with a mutation on the second *CYBB* allele outside the sequenced regions or the patient’s cells could have had an extremely skewed X chromosome inactivation pattern. An example was described before a female with a de novo mutation in gp91-phox coinciding with an extreme X chromosome inactivation ratio resulted in X-linked CGD [8]. However, X chromosome inactivation pattern was not assessed due to the lack of recipient’s blood stored before HSCT. At present, X chromosome inactivation pattern might be performed on other somatic tissues, but the parents are not willing to do any more tests. Mutations in *CYBB* have been documented in multiple patients with X-CGD [9]. Patients with CGD usually present with recurrent or severe infections; the most common sites are the lungs, skin, and lymph nodes [10]. In our case, a mutation was identified in our patient, who presented with disease onset at a young age. The clinical manifestations were recurrent hematochezia and abdominal pain, and abnormal findings were found during the diagnostic workup (respiratory burst test and immunoglobulin test). The patient had a history of lymph node abscess prior to admission, and the white blood cell count was slightly higher, although the inflammatory markers were within normal range, probably related to antibiotic use prior to admission at our institution. Indeed, *CYBB* deficiency is an X-recessive disease, usually not clinically expressed in females. However, it is also plausible that common single nucleotide polymorphism in *CYBB* alter the expression or function of gp91-phox, determining differences in susceptibility to complex disorders such as autoimmune or infectious diseases [11]. In contrast, Shahram and colleagues [12] reported a case with a mutation at the same site. The male child with consanguinous parents was diagnosed with chronic granulomatosis when he was 3 years old. He developed recurrent infections such as disseminated bacillus Calmette-Guérin (BCG) infection, otitis media, perianal abscess, pneumonia, and pulmonary abscess. The respiratory burst test showed the inability of phagocytes to generate ROS. He also had a maternal cousin with CGD. Therefore, *CYBB* mutations lead to clinical heterogeneity. Interestingly, although mutations of the same gene and site yield different clinical phenotypes, similarities prevail, including elevated inflammatory markers, abscess formation and dysfunction of phagocytes.

VEO-IBD accounts for 6-15% of children’s IBD [13]. Recent epidemiologic evidence suggests that the incidence of pediatric IBD is increasing, especially in VEO-IBD [14]. Early diagnosis is often challenging since the symptoms are atypical with younger age. Our case was initially misdiagnosed as cow’s milk protein allergy, leading to delayed diagnosis and treatment. It has been established that IBD has a multifactorial pathogenesis. Recent studies have suggested that VEO-IBD is associated with monogenic mutations [15]. Currently, IL-10 receptor deficiencies are the most common in China, and patients often present with severe ileocolonic inflammation and are often complicated with severe anal fistula [16]. A study in China [17] reported 39 cases of infant IBD, including 33 cases (85%) with moderate or severe disease activity index scores, resulting in 10 cases of death during the neonatal period. Accordingly, emphasis should be placed on avoiding misdiagnosis of allergies or infections in cases with recurrent diarrhea, hematochezia and malnutrition, especially those with early-onset age and VEO-IBD should be suspected. Indeed, further research is warranted to elucidate the features and mechanisms of this disease to improve the diagnostic and therapeutic efficacy rates.

Current evidence suggests that HSCT can heal intestinal mucosa, relieve clinical symptoms, and restore mitochondrial activity in phagocytes [18], consistent with our findings. HSCT represents a definitive cure for patients with *CYBB* mutations and IL-10 receptor deficiencies [19]. However, HSCT in IBD patients with *IKBKG* and *TTC7A* mutations can cause GVHD, severe infection, and even intestinal atresia [20]. Therefore, to achieve accurate treatment, it is necessary to ascertain whether gene mutations are present in VEO-IBD patients before treatment.

Moreover, genetic syndromes, such as Turner syndrome, Down syndrome, and glycogen storage disease type Ib, mainly characterized by chromosomal abnormalities, can present with IBD or are at high risk of developing IBD [21]. Therefore, the diagnosis should not be limited to clinical, endoscopic and pathological findings for patients with recurrent gastrointestinal symptoms in the early postnatal period. Indeed, a genetic analysis should be conducted to determine monogenic mutations, and early diagnosis and treatment should be performed. Importantly, HSCT can achieve a good therapeutic effect for patients with VEO-IBD caused by *CYBB* gene mutation. Our study contributes to the understanding of the rare disease and provides the foothold for further studies to improve the long-term prognosis of this patient population by improving the diagnosis and treatment efficacy rates. Only one patient was discussed in the present study. Further studies with more patients and longer follow-ups are warranted to improve current knowledge on such rare diseases.

## Data Availability

The datasets generated and/or analysed during the current study are available in the Genome Sequence Archive(GSA) for human repository with accession number : HRA004357 (https://ngdc.cncb.ac.cn/search/?dbId=hra&q=HRA004357). But the data isn’t release now, we provide with reviewer link (https://ngdc.cncb.ac.cn/gsa-human/s/i7Q29AdB).

## Abbreviations

IBD: Inflammatory bowel disease
VEO-IBD: Very early-onset inflammatory bowel disease
HSCT: Hematopoietic stem cell transplantation
X-CGD: X-linked chronic granulomatosis
DHR: Dihydrorhodamine
NADPH: Nicotinamide adenine dinucleotide phosphate
UC: Ulcerative colitis
CD: Crohn’s disease
ROS: Reactive oxygen species
NF-ĸB: Nuclear factor kappa-B
IgG: Immunoglobulin G
IgM: Immunoglobulin M
IgE: Immunoglobulin E
GVHD: Graft-versus-host disease
